# Multistable and multistep dynamics in neutrophil differentiation

**DOI:** 10.1186/1471-2121-7-11

**Published:** 2006-02-28

**Authors:** Hannah H Chang, Philmo Y Oh, Donald E Ingber, Sui Huang

**Affiliations:** 1 Vascular Biology Program, Department of Pathology and Surgery, Children's Hospital and Harvard Medical School, Boston, Massachusetts 02115, USA; 2 Program in Biophysics, Harvard University, Boston, Massachusetts 02115, USA

## Abstract

**Background:**

Cell differentiation has long been theorized to represent a switch in a bistable system, and recent experimental work in micro-organisms has revealed bistable dynamics in small gene regulatory circuits. However, the dynamics of mammalian cell differentiation has not been analyzed with respect to bistability.

**Results:**

Here we studied how HL60 promyelocytic precursor cells transition to the neutrophil cell lineage after stimulation with the differentiation inducer, dimethyl sulfoxide (DMSO). Single cell analysis of the expression kinetics of the differentiation marker CD11b (Mac-1) revealed all-or-none switch-like behavior, in contrast to the seemingly graduated change of expression when measured as a population average. Progression from the precursor to the differentiated state was detected as a discrete transition between low (CD11b^Low^) and high (CD11b^High^) expressor subpopulations distinguishable in a bimodal distribution. Hysteresis in the dependence of CD11b expression on DMSO dose suggests that this bimodality may reflect a bistable dynamic. But when an "unswitched" (CD11b^Low^) subpopulation of cells in the bistable/bimodal regime was isolated and cultured, these cells were found to differ from undifferentiated precursor cells in that they were "primed" to differentiate.

**Conclusion:**

These findings indicate that differentiation of human HL60 cells into neutrophils does not result from a simple state transition of a bistable switch as traditionally modeled. Instead, mammalian differentiation appears to be a multi-step process in a high-dimensional system, a result which is consistent with the high connectivity of the cells' complex underlying gene regulatory network.

## Background

**Figure 1:**
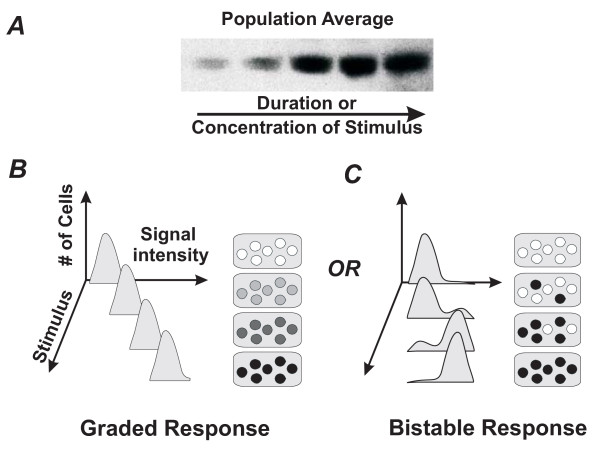
**Schematic illustration of how population measurements, such as Western blotting (*A*) cannot distinguish between graded (*B*) versus discrete responses (*C*)**. Sample cell population show gradual increase in marker expression as indicated by increasing hue (*B*) or switch-like response with cells either expressing or not expressing a marker upon stimulation (*C*). Flow cytometry histograms reveal the difference: a graded response would appear as one peak gradually shifting in intensity (*B*) whereas a bistable response would lead to two distinct peaks that alternatively grow or wane (*C*). "Stimulus" indicates duration of stimulation or concentration of stimulant.

During cell differentiation, an immature unspecialized cell assumes a new, stable and lasting phenotype [1]. Such a drastic change of cell identity is often considered to be a continuous process in which a precursor cell appears to gradually "morph" into a differentiated one. This impression arises in particular when expression of a specific differentiation marker is measured in a population of cells (e.g., using RT-PCR or Western blots) and is observed to gradually change over time after stimulation or as a function of the doses of the stimulus [2], as shown schematically in Fig. 1A,B. But in reality, the same continuous population-level change of marker expression can also arise if individual cells undergo an all-or-none "switch" into the differentiated state that occurs asynchronously (Fig. 1C). In fact, early developmental biologists recognized that cell phenotype "switches" may be discrete [3,4], but this perspective was lost as biochemical analysis of large populations of cultured cells came to dominate biology. Only with the advent of advanced methods for monitoring protein expression in individual cells has the notion of discontinuous switching between cellular states been revived. In these recent studies, increasing the dose of a stimulus has in fact been shown to increase the proportion of cells that make the transition from one state to another [5-9].

**Figure 2:**
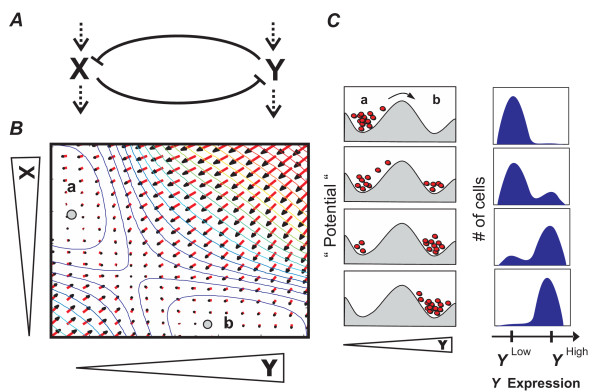
**Bistable dynamics in a two-gene system with cross-regulation**. *A*. Gene regulatory circuit diagram. Blunt arrows indicate mutual inhibition of genes *X *and *Y*. Dashed arrows indicate a basal synthesis (affected by the inhibition) and an independent first-order degradation of the factors. *B*. Two-dimensional XY phase plane representing the typical dynamics of the circuit. Every point (X, Y) represents a momentary state defined by the values of the pair X, Y. Red arrows are gradient vectors indicating the direction and extent that the system will move to within a unit time at each of the (X, Y) positions. Collectively, the vector field gives rise to a "potential landscape", visualized by the colored contour lines (numerical approximation). In this "epigenetic landscape", the stable states (attractors) are in the lowest points in the valleys: *a *(X>>Y) and *b *(Y>>X) (gray dots). *C*. Schematic representation of the epigenetic landscape as a section through *a *and *b *in which every red dot represents a cell. Experimentally, this bistability is manifested as a bimodal distribution in flow cytometry histograms in which the stable states *a *and *b *appear as peaks at the respective level of marker expression (e.g., Y).

Attempts to understand this all-or-none switching between phenotypes led to the reemergence of the concept of bistability. First proposed by Delbrück in 1948 [10] and later by Monod and Jacob [11] to explain differentiation, bistability describes how certain small regulatory circuits composed of one or two interacting genes can under certain conditions exhibit two and only two distinct equilibrium states. In a bistable system, the equilibrium states are relatively stable with respect to random perturbations imposed on the system [12]. However, conditions which give the system a large enough "push" can lead to a transition from one equilibrium state to the other. An example is the simple regulatory circuit illustrated in Fig. 2 consisting of two cross-inhibiting and spontaneously decaying genes or proteins, *X *and *Y*, which for appropriate interaction parameters can be mathematically shown to have only two stable equilibrium states in the two-dimensional X-Y state space: state *a *where (X>>Y) and state *b *where (Y>>X) (Fig. 2B). Since these are the *only *possible stable states of the X-Y circuit, the system can exhibit bistability with switch-like transitions between these two states [12]. These transitions are manifested as all-or-none switching between relatively persistent phenotypes when analyzed within single cells (Fig. 2C). Bistability also implies that under certain conditions, both equilibrium states are occupied simultaneously by the cells within one population. This type of behavior has been shown to arise in a variety of small gene regulatory circuits [12,13] in living organisms, including *Escherichia coli *[7,8] and *Saccharomyces cerevisiae *[6], as well as in signal transduction modules involving MAPK [9] and JNK [5] in *Xenopus *oocytes.

It is commonly postulated that bistability governs cellular differentiation in mammalian cells [14-16] athough the underlying genetic regulatory networks there are much more complex, but this has never been demonstrated experimentally. Instead of constructing artificial networks to exhibit bistability [6-8,15], we examined the validity of the bistable model in the context of mammalian differentiation by carrying out single cell analysis of human HL60 promyelocytic cells that are chemically induced to differentiate into neutrophils by treatment with dimethyl sulfoxide (DMSO). These studies show that a surface marker for differentiated neutrophils, CD11b (Mac-I) [17], is expressed in an all-or-none manner within individual cells, whether analyzed over time or in response to different levels of stimulus. However, detailed kinetic studies of the transition rate suggest that mammalian cell-fate switching may not simply be a bistable transition. Instead, differentiation appears to be a more complicated multistep process, a result which is consistent with the complexity of the underlying gene regulatory network which extends beyond the two-gene circuits used to model bistability.

## Results and discussion

### Bistability and bimodality

The human promyelocytic HL60 cells robustly differentiate into neutrophils within 6 days in the presence of 1.25% (v/v) DMSO [18], reaching stationarity with 50–70% of cells in the differentiated state as evaluated by morphological, biochemical, and molecular markers (see Material and Methods). We studied differentiation in HL60 cells by monitoring the expression of CD11b (Mac-1), a well-established surface marker for differentiated neutrophils [17]. Western blotting was used to measure the expression of CD11b as a population average, and in parallel flow cytometry was used to resolve expression of CD11b at the level of individual cells in the same population. Although the latter measurements are common place, a detailed kinetic analysis has not been reported previously.

**Figure 3:**
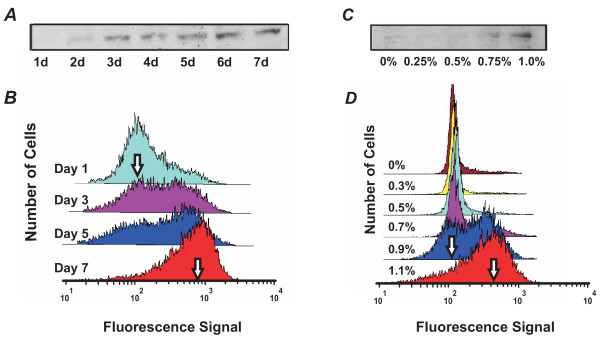
**HL60 differentiation exhibits bimodal response to both the duration and concentration of stimulant**. HL60 cells were cultured in 0.75% DMSO for 1 to 7 days (d) (*A*,*B*) or exposed to different doses (0% to 1.1%) of DMSO for 7 days (*C*,*D*) and monitored by Western blotting (*A*,*C*) and flow cytometry (*B*,*D*). Western blot analysis of whole cell populations revealed a gradual increase in intensity of the CD11b band both over time (*A*) and in response to increasing concentrations (*C*) of DMSO. In contrast, single cell analysis using flow cytometry demonstrated bimodality, as indicated by a shift of "peak heights" of the CD11b^Low ^(left arrows) and CD11b^High ^(right arrows) subpopulations in the histogram with increasing time of treatment (*B*) or dose of DMSO (*D*).

To determine whether CD11b expression in HL60 cells stimulated to differentiate into neutrophils by DMSO undergo discontinuous switching with a bimodal population distribution at intermediate stages that is characteristic of bistability, we monitored the time- and concentration-dependence of CD11b expression. As expected, when the whole population was analyzed using Western blots, CD11b expression appeared to increase gradually with increasing duration of DMSO treatment as the stimulated cells differentiated into neutrophils (Fig. 3A). In contrast, analysis of the underlying dynamics of CD11b expression at the individual cell level using flow cytometry revealed a bimodal distribution of low (CD11b^Low^) and high (CD11b^High^) CD11 expressor cells (Fig. 3B). As the duration of DMSO treatment increased from 1 to 7 days, the CD11b^High ^subpopulation grew, while the CD11b^Low ^subpopulation waned. A bimodal histogram in the flow cytometry signal representing roughly equal proportions of CD11b^High ^and CD11b^Low ^cells was observed at day 3. These results support a switch-like process in which the probability that individual cells will transition from the CD11b^Low ^state to the CD11b^High ^state increases upon treatment with DMSO. Even if bimodality was partially concealed by the overlap of the two subpopulations, the increased spread of the histogram during intermediate differentiation states, followed by its subsequent decrease as cells entered the differentiated state (Table 1) excludes the possibility of gradual differentiation kinetics.

**Table 1:** Mean and inter-quartile ratio (IQR) of fluorescence intensities for Fig. 3.

**Figure 3B**	Day 1	Day 3	Day 5	Day 7		
Mean	123.2	229.9	276.2	541.3		
IQR	5.00	9.67	11.32	3.86		

**Figure 3D**	0% DMSO	0.3% DMSO	0.5% DMSO	0.7% DMSO	0.9% DMSO	1.1% DMSO

Mean	197.1	138.4	170.7	282.9	291.1	395.7
IQR	1.37	1.25	1.41	3.12	4.24	3.59

The inter-quartile ratio was calculated as the ratio of the 75^th ^and 25^th ^percentiles in signal intensity for each time point in Fig. 3B and each DMSO concentration in Fig. 3D, representing the spread of the histogram.

Moreover, when we carried out similar studies in which we stimulated HL60 precursor cells with different concentrations of DMSO (0% to 1.1%) and analyzed CD11b expression at the stationary state (7d), we also observed bimodality (Fig. 3C, D). Again, analysis of the whole population using Western blots showed a gradual increase in CD11b expression with increasing DMSO concentration (Fig. 3C), whereas flow cytometry histograms of single cells demonstrated bimodality of the CD11b signal at 0.9% and 1.1% DMSO (Fig. 3D). Again, if the bell-shaped histogram of CD11 expression level shifted from low to high intensity values while maintaining its overall shape, it would indicate a gradual switching kinetics at the single cell level (Fig. 1B); however this was not observed (Fig. 3B, D). As in the time-course experiment, the non-monotonic evolution of the spread (Table 1) excludes the possibility of graded differentiation kinetics in individual cells.

The "blurring" of the two peaks that we observed in the bimodality (Fig. 3B,D) may be due to inherent population heterogeneity (e.g. due to stochasticity of gene expression [19]) which would lead to partial overlapping of the CD11b^Low ^and CD11b^High ^peaks, but does not invalidate the underlying switch-like kinetics.

### Hysteresis

To confirm that the observed bimodal response in CD11b expression was due to switch-like dynamics, rather than an ultrasensitive transition (i.e., steep step in the dose-response curve that acts a threshold [20]), we explored whether HL60 cells exhibit hysteresis in their CD11b expression response. Hysteresis implies a history-dependence of the response to the same stimulus and is a unique characteristic of a bistable system [21]. Here, hysteresis would manifest in the shape of the dose-response curve measured at stationary states for each DMSO dose, such that a stepwise reduction of the stimulus strength would produce a "lagging" of the corresponding decrease in response strength when compared to the dose-response curve obtained by increasing the strength of the stimulus.

Cells were first stimulated with increasing concentrations of DMSO for 7 days, reaching steady-state ("forward reaction"), and the proportion of cells that became CD11b^High ^was recorded. For the "backward reaction", maximally differentiated cells (treated with 1.25% DMSO for 7 days) were resuspended with various concentrations of DMSO for another 7 days to arrive at new stationary states, and the fraction of CD11b^High ^cells was similarly noted. Care was taken to ensure that the cells were never exposed to normal medium (except for the 0% DMSO backward reaction data point), because it cannot be excluded that such a short pulse of DMSO-free treatment may create unexpected and lasting effects interfering with hysteresis.

**Figure 4:**
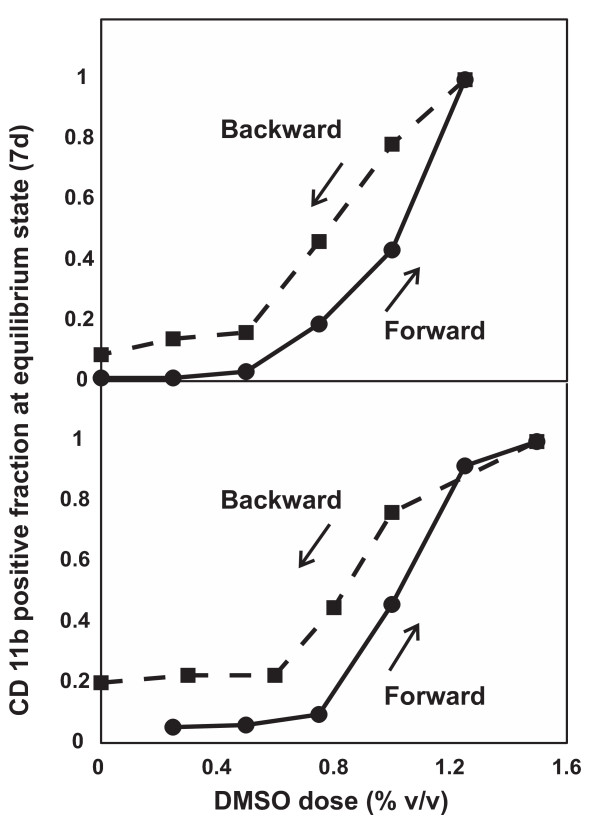
**DMSO-induced HL60 differentiation exhibits hysteresis**. HL60 cells were stimulated with 0% to 1.25%v/v DMSO (solid "forward" curve) for one week, after which cells in the 1.25% sample were washed and restimulated again in 0% to 1.25% DMSO (dashed "backward" curve). For both responses, differentiation was monitored by flow cytometry for CD11b after 7 days of stimulation (i.e. at stationary state). Vertical axis represents the fraction of CD11b^High ^cells relative to that exhibited by maximally differentiated cells exposed to 1.25% DMSO for 7 days. Note that the proportion of CD11b^High ^cells at a given DMSO concentration depended on the history of previous treatment ("forward" vs. "backward"). Duplicate results are presented; each data point represents a flow cytometry measurement obtained from 10,000 cells.

We observed a lag in cellular response in the backward versus forward reactions, as delineated by the two non-overlapping arms in the dose-response curves (Fig. 4) which is a hallmark of hysteresis [21]. It is important to note that the "retro-differentiated" cells re-assumed cell proliferation, hence increasing their proportion in the population. This effect diminishes the hysteresis loop by reducing the CD11b^High ^fraction in the cell cultures contributing to the backward reaction. Hence, the extent of the hysteresis loop is likely underestimated. The leftward shift of the backward dose response curve by approximately 0.2% cannot be explained by residual DMSO since suspension of the pellet (< 1 ul) into the medium (2 ml) before the reaction would have diluted the maximal concentration of 1.25% by greater than 1000 fold. Furthermore, the reappearance of the non-differentiated cells after the "backward reaction" cannot be attributed to regrowth of a small pool of DMSO-resistant cells because it was still observed to occur with similar kinetics when the CD11b^High ^subpopulation was isolated by FACS sorting before starting the "backward" treatment (not shown). In fact, a similar loss of differentiation characteristics (surface marker, enzymatic activity, etc.) has been observed in HL60 cells induced by 1α, 25-dihydroxyvitamin D_3 _into monocytes that were later removed from treatment [22].

The demonstration of bimodality and hysteresis in CD11b expression of differentiating HL60 cells is consistent with an underlying gene regulatory network of the structure shown in Fig. 2 that can give rise to bistable behavior. Such an architecture has been found in the regulatory circuit of transcription factors implicated in neutrophil differentiation involving the transcription factors GATA2 (= *X*) and PU.1 (= *Y*) which mutually inhibit each other [23-25]. For this well-studied system, a relatively broad range of interaction strength and stability of these factors gives rise to two equilibrium states [7,12,26,27]: state *a *in which GATA2 expression is high and PU.1 is low, and conversely, state *b*, in which PU.1 is high and GATA2 is suppressed. Biologically, state *b *may represent the differentiated neutrophil state because endogenous or enforced PU.1 upregulation activates the expression of many neutrophil specific genes, including the surface marker CD11b [28,29]. In contrast, state *a *represents the progenitor cell with low PU.1 and, in the case of the HL60 cells, higher levels of GATA2 [30]. Although artificially isolated as a module from a larger network, this small two-gene circuit captures the observed discreteness of a cell fate "switch" from the progenitor to the differentiated state. However, because the molecular targets of DMSO remain unknown, we cannot formally demonstrate how the hysteresis with respect to varying doses of DMSO arose; it only provides phenomenological support for bistability.

### Multi-step kinetics

Bistability has been proposed as a generic principle that governs differentiation in higher metazoans. However, since mammalian cell differentiation is controlled by regulatory interactions between hundreds, perhaps thousands of genes, and not by isolated one- or two-gene modules as widely assumed in bistability models [31], the multi-dimensionality of the switch-dynamics may be concealed by measuring a single variable. Using computational models, it has been previously shown that high-dimensional equivalents of bistability can exist in large genetic networks [32-38]. In these 'multi-stability' models, given some architectural constraints of the network, multiple equilibrium states, or "attractor states" may co-exist in high-dimensional state space. Experimental evidence for the existence of such high-dimensional attractor states that represent differentiated phenotypes has recently been shown in populations of HL60 cells using DNA microarray-based dynamic gene expression profiling [39]. Thus, we next examined whether the dynamics of switching in individual HL60 cells harbor evidence of multiple dimensions that could be revealed by monitoring a single variable (CD11b).

**Figure 5:**
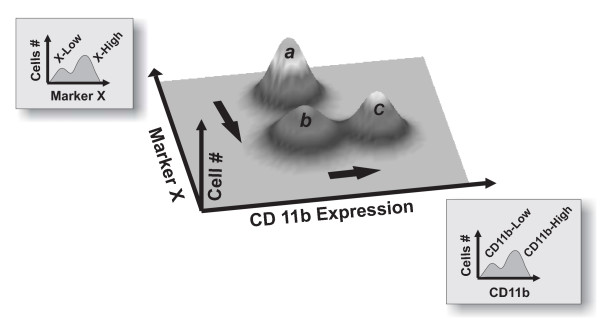
**Multistability in multiple state space dimensions**. Subpopulations identified as homogeneous with respect to a single marker (i.e., CD11b) may be heterogeneous with respect to another, unmeasured marker (e.g., Marker X). The arrows show a hypothetical path of differentiation whereby cells first change marker X expression (*a *to *b*) and then CD11b expression (*b *to *c*). An isolated CD11b^Low ^subpopulation thus may contain two (meta)stable states with respect to marker X (i.e. states *a *and *b*) that project into the same value with respect to CD11b.

Due to the abundance of mutual feedback regulation loops in the mammalian gene regulation network [26], it is possible that cells also undergo switch-like transitions in state space dimensions other than those linked to the expression of CD11b. However, the switching events in these other state space dimensions may not be synchronized. Consequently, a given subpopulation identified solely as CD11b^Low ^may be expected not only to differ from other members of the native (untreated) population, but also to be heterogeneous in composition, perhaps containing cells in multiple meta-stable states (Fig. 5). Since the genes spanning these other relevant state space dimensions are not known, existence of such metastable states may be detected as intermediate stages in the progression of differentiation. To explore this possibility, we compared native (untreated) CD11b^Low ^cells to CD11b^Low ^expressor cells isolated from a bimodal population produced by treatment with a submaximal dose (0.8%) of DMSO for 7 days. Specifically, we examined their responsiveness to a second-round of stimulation with 0.8% DMSO.

**Figure 6:**
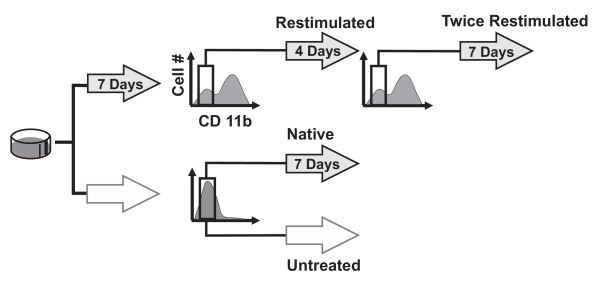
**Experimental design for analyzing multi-dimensional dynamics of differentiation**. Shaded block arrows represent cells exposed to 0.8% DMSO for the indicated durations while open arrows indicate "untreated" cell cultures. Brackets indicate subpopulations sorted with FACS. The fraction of CD11b^High ^cells was monitored by flow cytometry daily for 7 days for the "restimulated", "native" (mock-sorted and restimulated) and "untreated" groups. A CD11b^Low ^fraction that was re-established upon restimulation with 0.8% DMSO for 4 days was also sorted, restimulated a second time ("twice restimulated"), and monitored by flow cytometry for 6 days.

Seven days of DMSO treatment at 0.8% put the cell population in the bistable regime, in which typically more than half of the cells are in the CD11b^High ^state. The CD11b^Low ^cells were isolated using FACS, immediately recultured, and restimulated with 0.8% DMSO for another 7 days. During this period, flow cytometry analysis was performed daily to monitor the fraction of CD11b^High ^expressing cells (Fig. 6). In parallel, untreated native cells were mock-sorted and handled in exactly the same way as the sorted CD11b^Low ^subpopulation for comparison of the kinetics of differentiation into CD11b^High ^cells.

Three possible outcomes could be expected. First, the sorted CD11b^Low ^subpopulation could display a *decreased rate *of generating CD11b^High ^cells compared to the native mock-sorted cells. This outcome would indicate that the CD11b^Low ^subpopulation consisted of cells that were inherently more resistant to DMSO induction than native cells. In this case, the coexistence of both states in the bimodal culture would be due to heterogeneity of intrinsic responsiveness to the differentiation stimulus and selection, rather than from bistability as proposed in the bistable switch model of differentiation [6-9]. Second, the sorted CD11b^Low ^subpopulation could have the *same differentiation kinetics *upon restimulation with DMSO as the native cells. This would indicate that the CD11b^Low ^subpopulation of the bimodal distribution consisted of cells that "by chance" had not yet differentiated [40]. This possibility would not only support a simple bistable switch but also indicate that the state transition is a purely stochastic process. Stochasticity is often observed in cell fate choice and transitions in multipotent progenitor cells or stem cells [41-43], and could be related to the probabilistic manner by which cell type-specific genes are regulated by cis-regulatory elements [44,45]. The third and last possibility is that the sorted CD11b^Low ^subpopulation exhibits an *increased rate *of producing CD11b^High ^cells. This result would point to some "additive" effect of the two rounds of stimulation with DMSO wherein the first stimulation leads to progress in differentiation that is "stored" in state space dimensions other than CD11b.

**Figure 7:**
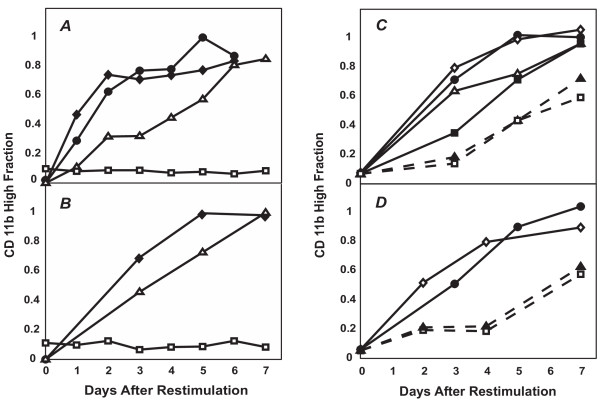
**The experiments outlined in Fig. 6 revealed that CD11b expression in DMSO-treated HL60 cells is a multi-step process involving an intermediate "primed" state**. (*A*,*B*). The "restimulated" (solid diamonds) and "twice restimulated" (solid circles) cells both showed a similar increased rate of CD11b expression (fraction of CD11b^High ^cells) relative to the "native" (open triangles) or "untreated" (open squares) groups. (*C*,*D*). This accelerated kinetics was sustained for up to four days in DMSO-free medium but was gradually lost beyond four days. Duplicates are presented, where solid circles, open diamonds, open triangles, solid squares, and solid triangles (--) represent cells cultured for 1, 2(for *C*)/4(for *D*), 5, 6, and 7 days in control medium before restimulation with 0.8% DMSO, respectively. Open squares indicate cells of the native population that have never been previously treated with DMSO nor FACS sorted. Each data point represents a flow cytometry measurement obtained from 10,000 cells.

**Figure 8:**
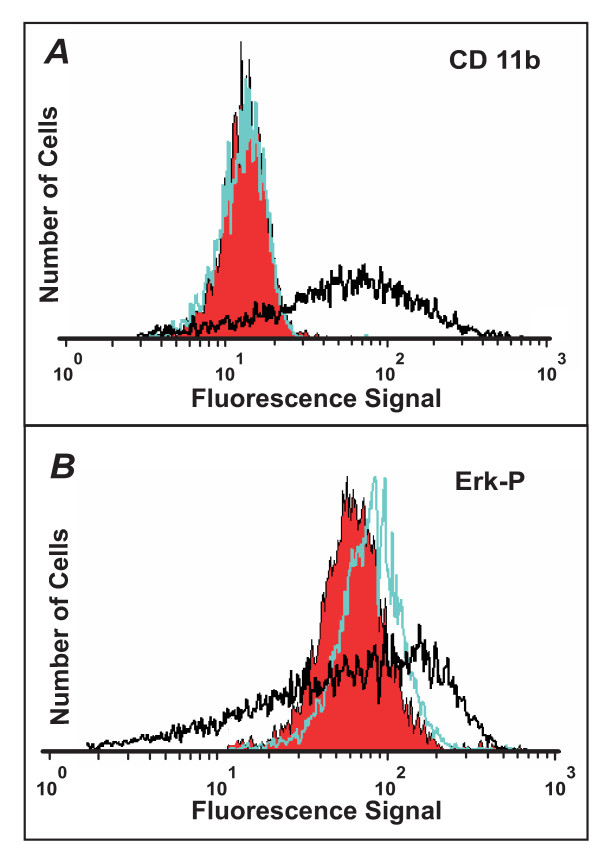
**The "primed" and native populations are indistinguishable by CD11b or phosphorylated-Erk expression levels**. *A*. The "primed" (green) and "native" (red) populations were measured for CD11b expression immediately after FACS sorting and found to be indistinguishable. For comparison, CD11b signal from the treated population before sorting is presented (black) showing a combination of CD11b^Low ^and CD11b^High ^cells. *B*. Similarly, intracellular phosphorylated-Erk (Erk-P) expression for "primed" (green) and "native" (red) populations as monitored by flow cytometry gave nearly identical results. In comparison, a sample treated with 1.3% DMSO for 7 days (black) revealed much larger variations in Erk-P expression.

We observed the last of the three possibilities: upon restimulation with intermediate-dose DMSO, the cells from the CD11b^Low ^subpopulation were not only capable of expressing CD11b, but did so at an accelerated rate compared to the native control population (Fig. 7A,B). It appears that the first seven days of exposure to intermediate-dose DMSO resulted in the "priming" of the CD11b^Low ^cells, in fact suggesting a 'metastable' intermediate state on the way to differentiation. Importantly, the primed status was not manifested in a change of CD11b (Figure 8A) or other known early markers of progressing differentiation, including loss of CD71 expression (not shown), or initial increase followed by loss of intracellular Erk phosphorylation (Fig. 8B) [46]. The level of phosphorylated-Erk (Fig. 8B) in the "primed" and "native" populations, as measured by flow cytometry immediately after FACS sorting were indistinguishable, as in the case of CD11b (Fig. 8A). Thus, Erk-phosphorylation changes cannot be used as a marker to differentiate between the "primed" and "native" populations. The "priming" process likely affects genes not monitored which in turn, may influence the rate of switching-on CD11b expression. Intriguingly, culturing these "primed" cells in normal (DMSO-free) medium for up to four days did not abolish the accelerated CD11b expression kinetics observed upon re-stimulation (Fig. 7C,D). This increased kinetics could not be attributed to the effects of residual intracellular DMSO since no increase in spontaneous differentiation was observed when the "primed" cells were placed in normal medium ("0 day" point in Fig. 7C,D). However, this memory was gradually lost with increasing time (> 4d) of culture in the absence of DMSO (Fig. 7C,D), supporting the metastable character of the primed state.

Interestingly, even after restimulating these cells (i.e., two rounds of DMSO in total), a CD11b^Low ^subpopulation was still observable. We thus asked the same question for the priming process as we originally did for the differentiation process: does the heterogeneity in the priming process result from the existence of other metastable "pre-primed" states or do the unprimed cells represent a resistant sub-population? To address this question, the CD11b^Low ^subpopulation that appeared after one round of re-stimulation with DMSO was FACS sorted and restimulated for a second time with 0.8% DMSO for another seven days (three rounds of DMSO treatment in total) (Fig. 6). Surprisingly, this subpopulation again showed accelerated CD11b expression kinetics upon restimulation when compared to the native control population (Fig. 7A), but had a rate of generating CD11b^High ^cells comparable to that exhibited by the subpopulation that was only exposed to DMSO for two rounds of stimulation (Fig. 7A). These results rule out the preexistence of a resistant subpopulation, and suggest that no additional intermediate steps between the CD11b^Low ^and the CD11b^High ^states can be discerned with the stimulation scheme used here.

Taken together, these results indicate that human HL60 cell differentiation is a multi-step process, consisting of at least two steps: (1) an initial transition step from the native CD11b^Low ^state to the "primed" CD11b^Low ^state and (2) a second step from the "primed" state to the CD11b^High ^state. The observation that a second round of sorting and restimulation did not alter the rate of CD11b^High ^cell production indicates that the process of "priming" (step 1) went through to completion (e.g. all "primed" cells are in the same state) at the perturbation strength conferred by 0.8% DMSO for 7 days. In contrast, the second step leading to the high expression of CD11b appeared to be a switch that only partially ran to completion in 0.8% DMSO, hence exposing not only the existence of a "primed" undifferentiated state, but also the stochastic nature of its transition to the differentiated CD11b^High ^state [47]. Given the design of our experiments, however, it was not possible to determine whether HL60 cells that are in the CD11b^High ^state must also pass through multiple sequential states to be fully differentiated with respect to all state space dimensions. Nevertheless, the existence of two discernible states (native and "primed") among the CD11b^Low ^subpopulation supports the existence of "deterministic heterogeneity" within this population as a result of multistability within the genome-wide regulatory network. This heterogeneity does not measurably contribute to additional population dispersion of CD11b expression levels in the CD11b^Low ^population. Instead, it represents an additional state in which the cells exhibit an increased readiness to express CD11b upon restimulation.

Our results may also explain why cellular differentiation processes often take as long as several days to weeks to complete, although molecularly, they essentially consist of a change in gene expression profile which could be completed in a day at the level of individual genes. Specifically, the results also explain why, despite hysteresis, prolonged exposure (> 3 days) to the stimulating agents DMSO and all-trans-retinoic acid is necessary to achieve maximal neutrophil differentiation in HL60 cells ([18,48]; H.H.C. and S.H., unpublished observations). This is in contrast to other HL60 differentiation processes, such as macrophage differentiation, for which brief exposure to TPA of a few hours is sufficient [49].

At the moment, the molecular mechanisms that establish the primed state are not known. It is likely that in order to switch the transcriptome to that of the differentiated state, multiple waves of transcriptional activation must occur in which newly synthesized transcription factors regulate other (transcription) factors. Thus, the primed state may reflect a state in which such intermediate regulatory proteins have become available. Moreover, remodeling of chromatin to make loci of differentiation-specific genes accessible for transcription may contribute to the multi-step characteristics of differentiation with kinetically identifiable intermediate states [50].

## Conclusion

In this study, we examined the dynamics of mammalian cell differentiation by studying the expression kinetics of the differentiation marker CD11b during neutrophil differentiation in DMSO-treated HL60 cells. Although the behavior of this marker is in agreement with simple bistability models [10,11], our detailed analysis revealed that differentiation is actually a multi-step process consistent with a model in which multiple coupled switches along various state space dimensions give rise to multistable states that represent high-dimensional attractors in the genome-wide cell regulatory network [33,39]. Based on a purely dynamical and phenomenological analysis, we were able to identify a "primed" state in HL60 differentiation characteristic of cells that had not made the all-or-none *phenotypic *switch, but had proceeded partially along the path of differentiation. Although we were unable to take a "bottom-up" approach as in the studies of well-characterized microorganisms [8] or engineered gene networks [7], our treatment strategy and sorting scheme allowed us to study mammalian cell differentiation without knowing the underlying gene regulatory network beyond the GATA2/PU.1 switch. This approach opens a new way of dissecting the multi-step process of cellular differentiation into a sequence of discrete metastable intermediate states that evade conventional time-course analysis of entire populations. Specifically, our results suggest that the regulation of differentiation may involve "unanticipated" gene dimensions which do not directly affect the expression of a measured marker. The existence of multi-dimensional, multistable behavior during cell fate switching in mammalian cells has important implications in the way differentiation is viewed and ultimately, in how processes such as lineage commitment of stem cells during tissue development can be explained and controlled.

## Methods

### Cell culture and differentiation

HL60 cells (ATCC) were cultured in IMDM medium (ATCC) supplemented with 10% fetal bovine serum and 1% glutamine plus penicillin and streptomycin. Cells of passage 7 (after receipt from ATCC) at a density of 1.0 × 10^6 ^cells/ml and growing at a basal rate of 1.3–1.7 day ^-1 ^were treated with variable concentrations of DMSO (Sigma) ranging from 0.3% to 1.25% (v/v) to induce differentiation. At each time point, cells were harvested from the suspension culture, pelleted, and processed for either Western blot and/or flow cytometry analysis (see below). Differentiation was monitored primarily with CD11b expression by flow cytometry, but morphology by Giemsa stain and nitroblue tetrazolium-reducing activities were also utilized. A stationary state was reached at day 6 since the fraction of differentiated cells and the level of expression of CD11b did not further increase when cells were monitored up to day 12 after induction of differentiation.

### Western blot analysis

1 × 10^6 ^cells were pelleted and directly lysed with 20% sample-loading buffer for SDS-polyacrylamide gel electrophoresis (SDS-PAGE) and immediately boiled for 5 min at 95°C. 30–50 μl of total cell lysate were fractionated on SDS-PAGE gel and transferred to nitrocellulose membranes. Following blocking with 5% milk/PBST (phosphate buffered saline with Tween 20), the membrane was probed with a 1:500 dilution of CD11b/Mac-1 antibody (BD Pharmingen). Antibody binding was detected with a 1:5000 dilution of peroxidase labeled anti-mouse IgG (Vector) and luminescence was detected with Supersignal West Dura Signal reagents (Pierce).

### Immunofluorescence staining of live cells for flow cytometry

For the Guava- PCA system (see below) 200,000 cells were pelleted and incubated in 7 μl of CD11b/MAC-I R-PE conjugated fluorescence antibody (BD Pharmingen) on ice for 30 min, washed with ice-cold 1% fetal calf serum/PBS/0.01% NaN_3 _(NaN_3 _is left out in sorting experiments), and resuspended in the same buffer at 10^6 ^cells/ml density for analysis. Intracellular phosphorylated-Erk levels were detected using the BD PhosFlow kit (BD Pharmingen) and the protocol provided. Briefly, 200,000 cells were fixed with BD PhosFlow Fix Buffer (BD Pharmingen) at 37°C for 10 min, pelleted, washed with BD PhosFlow Perm/Wash Buffer (BD Pharmingen) twice, incubated with 5 μl of a 1:5 dilution of Anti-Phospho-ERK1/2:PE conjugated fluorescence antibody (BD Pharmingen) in the dark at room temperature for 1 hour, washed again with Perm/Wash Buffer, and resuspended in the same buffer at 10^6 ^cells/ml density for analysis. For fluorescence-activated cell sorting, staining was scaled up 10-fold to 50 μl of CD11b/MAC-I R-PE conjugated fluorescence antibody (BD Pharmingen) per 10^6 ^cells and cells were resuspended at 8–10 × 10^6 ^cells/ml. Pilot antibody titration experiments were performed to ensure that staining occurred at least at 2-fold saturation. Ice-cold 1% fetal calf serum/PBS/0.01% NaN_3 _was used to establish background signal with unstained cells.

### Flow cytometry and Fluorescence Activated Cell Sorting (FACS)

Flow cytometry was performed on a Guava-PCA microfluidic-based flow cytometer (GuavaTechnologies, Inc). Fluorescence activated cell sorting was performed with either a Becton Dickinson FACSVantage (Becton Dickinson) or a Becton Dickinson FACSAria (Becton Dickinson) flow cytometer. Data analysis was done with either CytoSoft™ 2.1.1. (GuavaTechnologies, Inc) or WinMDI software. For cell sorting, starting cell number ranged between 40–80 × 10^6 ^cells, and cells were sorted into ice-cold medium for a maximum of 3 hours. Gates for sorting the CD11b^Low ^subpopulation in the 0.8% DMSO-treated samples were set relative to an untreated, native population. The latter was also mock sorted and processed in exactly the same way as the former to control for the effects of FACS sorting on cellular expression of CD11b. To remove the staining antibody before reculturing, pelleted cells were suspended in pH.2.25 MES (morpholinoethanesulfonic acid)/Tris buffer for 30 s. A 10-fold volume of pH 7.4 PBS was immediately added for neutralization and the cells were pelleted and resuspended in culture medium. After antibody removal the cells had fluorescence signal intensities on par with unstained HL60 cells and exhibited normal viability for future immunofluorescence staining.

## Authors' contributions

H.H.C. participated in the design of the study, performed the experiments, and drafted the manuscript. P.Y.O. established the antibody-removal protocol. D.E.I. supervised the work and revised the manuscript. S.H. conceived of the study, participated in the experiments, and drafted the manuscript. All authors read and approved the final manuscript.
